# Miscellaneous Special

**Published:** 1892-12-24

**Authors:** 


### MISCELLANEOUS. SPECIAL.

**Cancer Hospital**, Fulham Road, Brompton, S.W.—This free hospital, founded in 1851, shelters over six hundred sufferers, not only from this most painful disease, but also from many species of tumours which, if neglected, might prove fatal, but, if dealt with in time, are curable; it also benefits a large number of out-patients. The annual expenditure is about £3,000 in excess of the reliable income, to meet which new annual subscriptions and donations are urgently needed. Secretary, Mr. W. H. Hughes; Matron, Miss A. Rogers.

**Central London Throat and Ear Hospital**, Gray's Inn Road, W.C.—The hospital was established eighteen years ago for the treatment of poor patients suffering from diseases of the throat, nose, and ear. It is situated in a very poor and populous district, while its contiguity to the various railway termini at King's Cross renders it easily accessible for patients from all parts of London, the suburbs, and the country. The hospital has no endowment whatever, and is dependent entirely upon voluntrry contributions. Secretary, Mr. R. Kershaw; Matron, Miss M. Danter.

**Hospital for Diseases of the Throat**, Golden Square, W.—Owing to the very large proportion of children amongst the patients treated here, the Committee have, had their Board-room converted into a children's ward. The Committee have no funds to meet the additional expense thus entailed, and they earnestly hope that some portion of the charitable gifts of the season will be devoted to this object. Secretary, Mr. W. Thornton Sharp; Matron, Miss M. B. Mackey.

**Lock Hospital and Asylum**. Harrow Road, W.—Rescue work, as well as the cure of the patients who present themselves for admission, is the aim of this institution, and if it were more generally known what good work is being done by its means, we feel sure that it would meet with the support it deserves. The result, that considerably more than one-fourth of all those that pass through this hospital are reclaimed from their evil lives, points to a great work, which should be far more encouraged. A debt of £4,600 has still to be cleared off, and at least £1,500 is required in fresh annual subscriptions to meet the regular requirements of the institution. Matron of hospital, Miss Kydd; Matron of asylum, Miss J. McNiven.

**Royal London Ophthalmic Hospital**, Moorfields, E. C.—An urgent appeal is made by the Board of Management for aid in the erection of a new hospital on ground recently leased to them by the City of London, and in the re-arrangement of the old hospital adjoining, to meet the medical and sanitary requirements of the day. The lowest estimated cost of the new building and alterations alone is £35,000, exclusive of cost of refurnishing, and additional annual expenditure consequent on the increase in size. All patients are admitted free, and without letters of recommendation. Secretary, Mr. Robert J. Newstead; Matron, Miss Nichol.

